# Oligomeric Procyanidin Nanoliposomes Prevent Melanogenesis and UV Radiation-Induced Skin Epithelial Cell (HFF-1) Damage

**DOI:** 10.3390/molecules25061458

**Published:** 2020-03-24

**Authors:** Yashu Chen, Fenghong Huang, David Julian McClements, Bijun Xie, Zhida Sun, Qianchun Deng

**Affiliations:** 1 Oil Crops Research Institute, Chinese Academy of Agricultural Sciences, Wuhan 430062, China; yashuchen@sina.com (Y.C.); fhhuang@foxmail.com (F.H.); 2 Department of Food Science, University of Massachusetts Amherst, Amherst, MA 01003, USA; mcclements@foodsci.umass.edu; 3 Natural Product Laboratory, Department of Food Science and Technology, Huazhong Agricultural University, Wuhan 430070, China; bijunxie@sina.com (B.X.); sunzhida@sina.com (Z.S.)

**Keywords:** lotus seedpod oligomeric procyanidin, nanoliposomes, melanogenesis, UV-induced damage, skin aging

## Abstract

The potential protective effect of nanoliposomes loaded with lotus seedpod oligomeric procyanidin (LSOPC) against melanogenesis and skin damaging was investigated. Fluorescence spectroscopy showed that, after encapsulation, the LSOPC-nanoliposomes still possessed strong inhibitory effects against melanogenesis, reducing the activity of both monophenolase and diphenolase. Molecular docking indicated that LSOPC could generate intense interactive configuration with tyrosinase through arene–H, arene–arene, and hydrophobic interaction. An ultraviolet radiated cell-culture model (human foreskin fibroblast cell (HFF-1)) was used to determine the protective effects of the LSOPC-nanoliposomes against skin aging and damage. Results showed that LSOPC-nanoliposomes exerted the highest protective effects against both ultraviolet B (UVB) and ultraviolet A (UVA) irradiation groups compared with non-encapsulated LSOPC and a control (vitamin C). Superoxide dismutase (SOD) and malonaldehyde (MDA) assays demonstrated the protection mechanism may be related to the anti-photooxidation activity of the procyanidin. Furthermore, a hydroxyproline assay suggested that the LSOPC-nanoliposomes had a strong protective effect against collagen degradation and/or synthesis after UVA irradiation.

## 1. Introduction

The skin is the outermost physical barrier protecting humans from external injuries [1]. Sunlight induces the formation of electrons (e-) and holes (h+), whose interactions lead to the accumulation of reactive oxygen species (ROS), including superoxide anion radical (•O^2−^), singlet oxygen (_1_O^2^), and hydroxyl radicals (•OH), etc. [2]. High levels of ROS give rise to various skin damages, such as inflammation, wrinkling, discoloration, and erythema/edema [3]. The ultraviolet (UV) radiation in sunlight is believed to be the main cause of these adverse effects.

**Figure 1:**
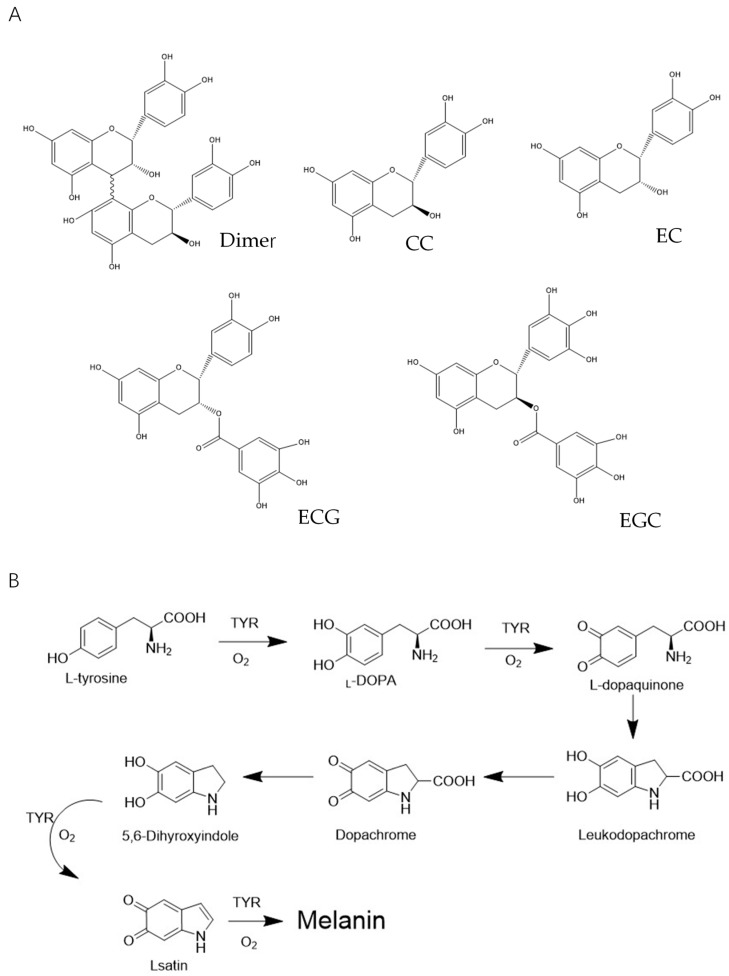
(**A**) The main compounds of lotus seedpod oligomeric procyanidin (LSOPC); (**B**) the key formation steps of melanin and the possible inhibition mechanism of LSOPC and LSOPC Nano.

The brownish polymer melanin in skin is produced in melanocytes, located on the basal layer between the dermis and epidermis. The type, amount, and distribution of melanin determines skin color. Melanin functions in humans similar to polyphenols, absorbing UV sunlight and removing ROS to protect the skin from UV damage. However, superfluous levels of melanin cause various dermatological disorders, including color spots, melasma, and other actinic damage [4]. Thus, there is considerable interest in the identification of natural source compounds as UV-protective materials. Lotus seedpod oligomeric procyanidin (LSOPC), a kind of B-type procyanidin, is a mixture of various molecules (Figure 1A) mainly containing catechin/epicatechin monomers, dimers, ECG, and EGCG [5]. This plant-based bioactive agent has previously been shown to exhibit a variety of beneficial health effects. In vivo and in vitro studies have demonstrated that it has hypolipidemia, hypoglycemic activity, and the ability to inhibit cognitive impairment [6,7,8,9]. Furthermore, Duan et al. [10] reported that procyanidins from lotus had significant growth inhibition effects on mouse melanoma B16 both in vivo (inhibiting tumor growth in C_57_BL/6 J mice by 55.3% in terms of average tumor weight) and in vitro (possessing cytotoxicity against mouse melanoma B16 cell line). Based on LSOPC’s multiple functions and enormous health benefits, products that apply LSOPC including capsules have been made and used as nutrient enhancers. However, there is still much potential for the application of LSOPC in the food industry.

However, the poor solubility, low chemical stability, and low bioavailability of LSOPC currently limit its application for skin protection. These challenges may be overcome by using colloidal delivery systems inspired by nanotechnology. In the food industry, this type of delivery system is already widely used to increase the solubility, stability, and bioavailability of bioactive food components [11]. A wide variety of different nanostructured delivery systems is available, including microemulsions, nanoemulsions, solid lipid nanoparticles, nanoliposomes, and biopolymer nanoparticles [12]. Each of these delivery systems has its own set of advantages and limitations. Nanoliposome is one of the most effective systems for encapsulating water-soluble materials and protecting these bioactive substances from oxidation, metal ions, pH variations, and enzyme-induced reactions [13,14,15,16]. In our previous studies, we prepared LSOPC-nanoliposomes (LSOPC Nano) using reverse-phase evaporation combined with sonication. The encapsulated LSOPC was found to have better stability against environmental stress and free radical scavenging activity than the free form [17]. However, how the encapsulation process (that might bury the active molecular group of LSOPC along with increased solubility and stability) would change its protective bioactivities against skin damage is unknown. To answer that unclear question was the main purpose of this study.

The synthesis of melanin in the skin involves a series of enzymatic and non-enzymatic reactions [18]. Tyrosinase, a copper-containing monooxygenase, is involved in the first step of melanin synthesis by catalyzing l-tyrosine to l-3,4-dihydroxyphenylalanine (l-DOPA). Thereafter, l-DOPA is rapidly converted into l-dopaquinone, again catalyzed by tyrosinase, and then undergoes several reactions to eventually form melanin (Figure 1B). As the level of substrates such as l-tyrosine and l-DOPA increases, the rate of these melanin-forming reactions increases [19]. Moreover, UV can directly stimulate the proliferation of melanocytes and increase melanin biosynthesis. Therefore, the inhibition of melanogenesis is generally evaluated when screening potential inhibitors of UV-induced skin damage. This typically involves monitoring the inhibition of both the tyrosinase (including monophenolase and diphenolase) and the substrates (l-tyrosine and l-DOPA). The inhibition effects of both LSOPC-nanoliposomes and LSOPC against melanogenesis were measured using a fluorescence method. Then, molecular docking was performed to explore the potential interaction between LSOPC and tyrosinase and the molecular mechanism of the anti-melanogenesis effect. Additionally, the potential cytotoxicity and protective effects of LSOPC Nano on a cell culture model (human foreskin fibroblast cell (HFF-1)) against UVA and UVB radiation were measured and compared with free LSOPC and a common water-soluble antioxidant (vitamin C). We further measured its potential protective mechanism using superoxide dismutase (SOD), malonaldehyde (MDA), and hydroxyproline assays. The objective of this study was to find out the potential effects of encapsulated LSOPC on preventing melanogenesis and UV radiation-induced skin epithelial cell (HFF-1) damage. The information obtained in this study may lead to the formulation of new plant-based skin treatments to protect against sun damage.

## 2. Results and Discussion

### 2.1. Residual Solvent Measurement in LSOPC-Nanoliposomes

Gas chromatography was used to determine the presence of any residual organic solvents in the LSOPC-nanoliposomes after their fabrication. Ethanol was used as an internal standard. According to Figure S1, the retention times of ethanol (peak 1) and chloroform (peak 2) were 2.67 and 3.30 min, respectively. There was no obvious peak in Figure S1C, which indicated that the level of chloroform present in the LSOPC-nanoliposomes was below the limit of detection.

### 2.2. Effects of LSOPC-Nanoliposomes on Monophenolase Activity

**Figure 2:**
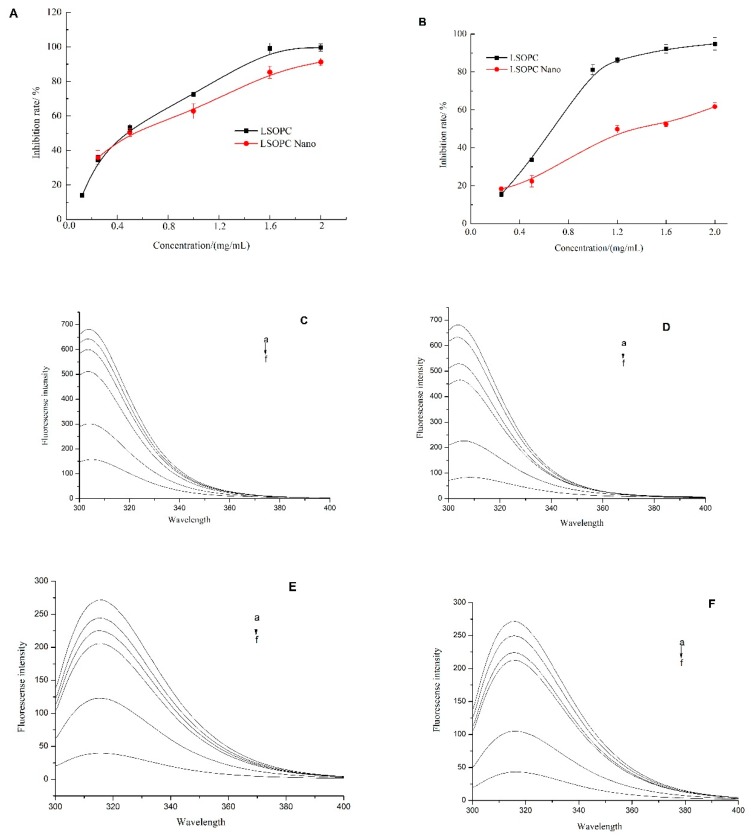
Inhibition effects of LSOPC and LSOPC Nano on monophenolase (**A**) and diphenolase (**B**), respectively. Fluorescence spectra of l-tyrosine in the presence of different concentrations of LSOPC (**C**) and LSOPC Nano (**D**). Fluorescence spectra of l-3,4-dihydroxyphenylalanine (l-DOPA) in the presence of different concentrations of LSOPC (**E**) and LSOPC Nano (**F**). (a)–(f): 0, 25, 50, 100, 250, and 500 μg/mL.

According to Figure 2A, LSOPC-nanoliposomes exhibited a strong inhibitory effect against monophenolase in a dose-dependent manner. For instance, at a level of 2 mg/mL, they inhibited monophenolase activity by 91.7%. The IC_50_ of the encapsulated LSOPC (0.64 ± 0.06 mg/mL) was slightly higher than that of the non-encapsulated LSOPC (0.56 ± 0.03 mg/mL). This relatively small difference indicates that the encapsulation process not only protects the stability of the LSOPC but also maintains its high inhibitory effects against monophenolase activity.

### 2.3. Effects of LSOPC-Nanoliposomes on Diphenolase Activity

According to Figure 2B, LSOPC and LSOPC-nanoliposomes both exhibited inhibitory effects against diphenolase in a dose-dependent manner. At a level of 2 mg/mL, the inhibition rates of diphenolase activity for the non-encapsulated and encapsulated LSOPC were 94.8% and 61.7%, respectively. The IC50 of the encapsulated LSOPC (1.46 ± 0.03 mg/mL) was nearly 2-fold greater than that of the non-encapsulated form (0.73 ± 0.02 mg/mL). This result indicates that encapsulation of the LSOPC did appreciably reduce its inhibitory effects against diphenolase activity. Fujimaki et al. [20] reported the inhibitory effect on melanin synthesis of proanthocyanidins from red wine. The fraction 7 of the proanthocyanidins isolation was found to have the strongest tyrosinase inhibitory activity, which had the highest oligomeric proanthocyanidin content, whereas fractions 1 and 2 with the high anthocyanin and low oligomeric proanthocyanidin content had weak activity. These results were consistent with our hypothesis that supports the potential application of LSOPC in anti-melanogenesis. However, research on the role of encapsulated oligomeric proanthocyanidin in melanogenesis seems to be lacking for the present. However, in another published article, liposome-encapsulated anthocyanin was reported to enhance DPPH scavenging activity and inhibitory effects on melanin production in vitro [21], which partially supports our results that liposome-encapsulated flavonoid LSOPC might still preserve a better related bioactivity.

### 2.4. Fluorescence Quenching Analysis

l-tyrosine and l-DOPA are two crucial intermediate products in the melanin formation process. A fluorescent assay was therefore used to determine the interaction of LSOPC and LSOPC-nanoliposomes with these two intermediate products. According to Figure 2C,D, the fluorescent intensity of l-tyrosine decreased when the concentration of both non-encapsulated and encapsulated LSOPC increased. Both LSOPC and LSOPC-nanoliposomes showed no red-shift or blue-shift effect on l-tyrosine, which indicates no obvious structural change in the l-tyrosine during the interaction. For the non-encapsulated systems, the fluorescent intensity of l-tyrosine decreased by 5.84% and 77.0% after interacting with 25 and 500 μg/mL of LSOPC, respectively. Similarly, for the encapsulated systems, the fluorescent intensity decreased by 7.23% and 87.8%. The IC50 of the LSOPC-nanoliposomes (226.6 ± 7.6 μg/mL) was smaller than that of the LSOPC (284.0 ± 9.7 μg/mL), which suggests encapsulation promoted the interaction of LSOPC with l-tyrosine.

According to Figure 2E,F, the fluorescent intensity of l-DOPA decreased when the concentration of non-encapsulated and encapsulated LSOPC increased. Both LSOPC and LSOPC-nanoliposomes showed no red-shift or blue-shift effect on l-DOPA, which suggests there was no major structural change of the l-DOPA as a result of the interaction. For the non-encapsulated system, the fluorescent intensity of l-DOPA decreased by 9.91% and 85.3% after interacting with 25 and 500 μg/mL of LSOPC, respectively. Similarly, for the encapsulated system, the fluorescent intensity decreased by 8.02% and 84.1%. The IC50 of the LSOPC-nanoliposomes (256.1 ± 7.2 μg/mL) was slightly smaller than that of the LSOPC (265.2 ± 5.2 μg/mL), which suggests that encapsulation somewhat facilitated the interaction of LSOPC with l-DOPA. 

Overall, these results show that the LSOPC-nanoliposomes possessed a higher affinity for the melanogenesis substrates than free LSOPC, which may indicate a higher preventive effect against skin spots.

### 2.5. Docking Studies

**Figure 3:**
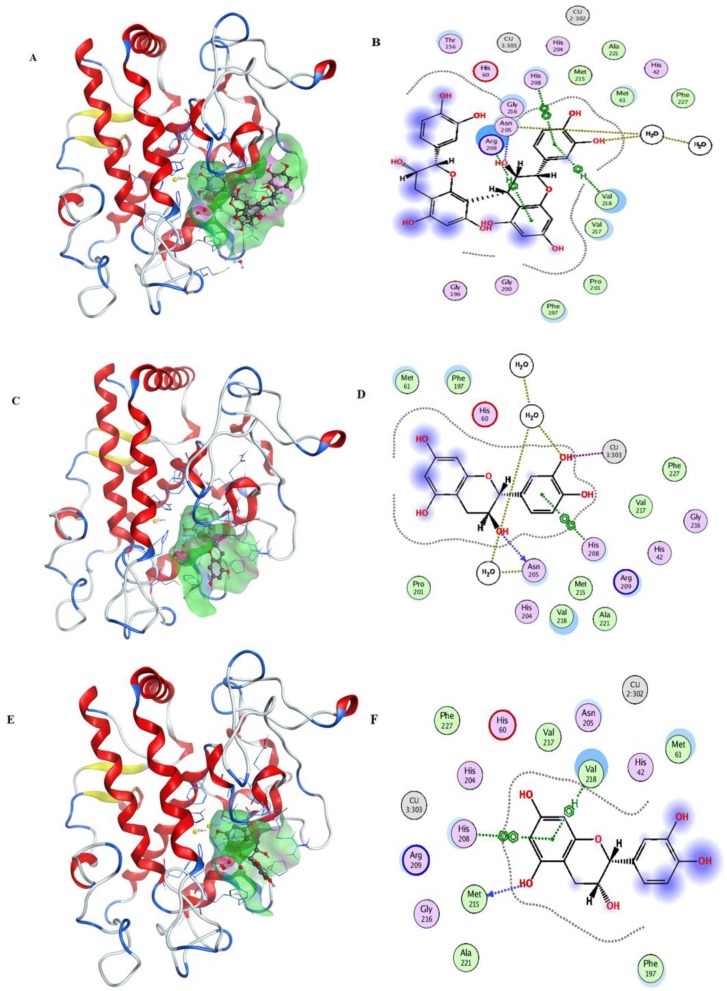

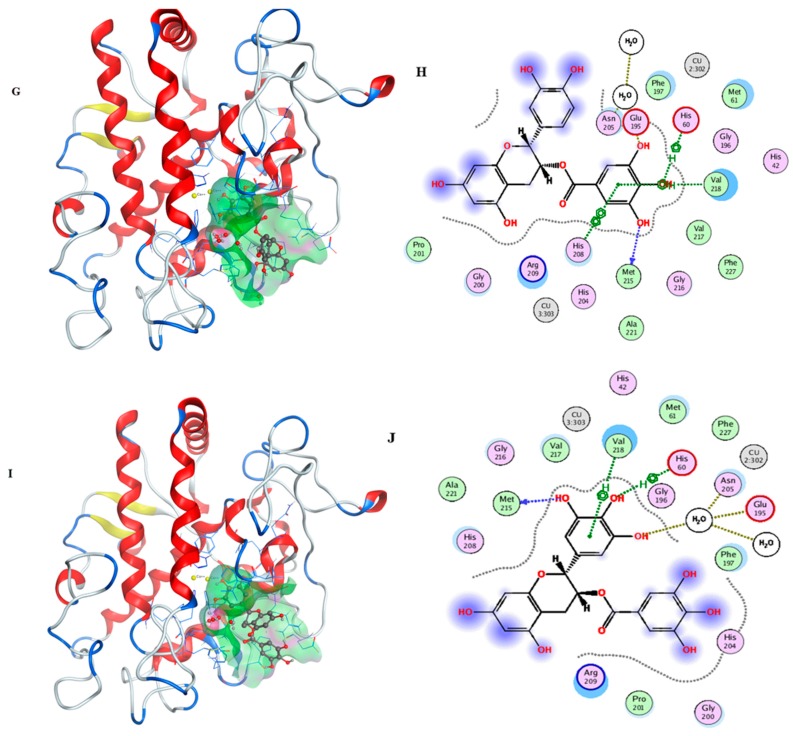
Ligands are shown as grey, site residues are shown as blue, copper ions are shown as yellow. (**A**) binding conformation of LSOPC dimer binding to the tyrosinase; (**B**) molecular contacts between LSOPC dimer and amino acids of tyrosinase; (**C**) binding conformation of compound CC binding to the tyrosinase; (**D**) molecular contacts between compound CC and amino acids of tyrosinase; (**E**) binding conformation of compound EC binding to the tyrosinase; (**F**) molecular contacts between compound EC and amino acids of tyrosinase; (**G**) binding conformation of compound EGC binding to the tyrosinase; (**H**) molecular contacts between compound EGC and amino acids of tyrosinase; (**I**) binding conformation of compound EGCG binding to the tyrosinase; (**J**) molecular contacts between compound EGCG and amino acids of tyrosinase.

After the docking simulation, the structure of the most likely binding conformation and the 2D interaction diagram of tyrosinase with ligands in site were captured. Figure 3A,B shows that the LSOPC dimer can fit into site and generate intense interaction with the tyrosinase protein. It is intended to generate arene–H interaction with tyrosinase residue Arg209 and Val218, respectively. Furthermore, it can form an arene–arene interaction with tyrosinase residue His208. The S-score of this binding conformation is −7.7110. Figure 3C,D shows compound CC can also perfectly fit into site. The residue His208 of tyrosinase is intended to generate an arene–arene interaction with compound CC. In particular, compound CC has an interaction with a copper ion, which is the catalytic activity center of tyrosinase. The S-score of this binding conformation is −6.8215. Figure 3E,F shows that compound EC can also fit into the docking site. The residue Val218 and His208 of tyrosinase are intended to generate an arene–H interaction and arene–arene interaction with compound EC, respectively. The S-score of this binding conformation is −5.9393. Figure 3G,H shows the interaction of ECG and tyrosinase. The residue Val218 and His60 of tyrosinase are intended to generate arene–H interactions with compound ECG, and His208 to generate an arene–arene interaction with compound ECG. The S-score of this binding conformation is −7.2141. Figure 3I,J shows the configuration of EGCG binding to tyrosinase. The residue Val218 and His60 of tyrosinase are intended to generate an arene–H interaction with compound EGCG, respectively. The S-score of this binding conformation is −7.0136. In summary, these typical LSOPC molecules can all bind to the protein tyrosinase nicely, and hydrophobic interactions were the major interaction force.

### 2.6. Cytotoxicity Assay

**Figure 4:**
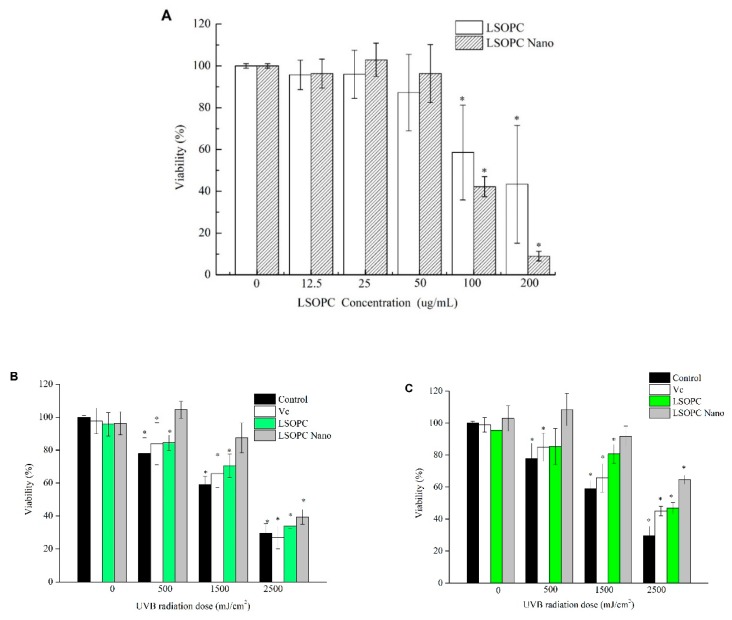
Cytotoxic effects of different concentrations of LSOPC and LSOPC Nano (**A**); protective effects of Vc, LSOPC, and LSOPC Nano on human foreskin fibroblast cell (HFF-1) after ultraviolet B (UVB) irradiation; the concentrations of Vc, LSOPC, and LSOPC Nano were as follows: (**B**) 12.5 μg/mL; (**C**) 25 μg/mL. * *p* < 0.05.

The effects of LSOPC and LSOPC-nanoliposomes on HFF-1 cell viability is shown in Figure 4A,B. At 50 μg/mL, neither the non-encapsulated nor the encapsulated form of LSOPC showed any suppression effect on the viability of HFF-1 cells. Conversely, at 100 μg/mL, both LSOPC and LSOPC-nanoliposomes exerted toxic effects on the HFF-1 cells.

### 2.7. Protective Effect on HFF-1 Cell Viability under UVB Irradiation

In the dermis of the skin, fibroblasts produce and deposit the collagen and elastic fibers that make up the extracellular matrix. Furthermore, fibroblasts are the major mesenchymal cell type in the connective tissue and play an important role in dermal architecture in both skin formation and repair [22]. The human foreskin fibroblast cell (HFF-1) is one of the main types of human fibroblasts. Many previous studies have focused on photoaging and skin cancer [1,23], while studies on the effects of UV radiation on HFF-1 are rare. In particular, the impact of UV on the viability of HFF-1 cells and the physiological alterations involved remain unclear.

The MTT assay was used to investigate the protective effects of the LSOPC-nanoliposomes on HFF-1 cells exposed to UVB irradiation. Compared to the non-irradiated cells, the cell viability of HFF-1 after exposure to 500 mJ/cm^2^ UVB irradiation was reduced to 77.9% (Figure 4A). When the UVB radiation was increased to 2500 mJ/cm^2^, the cell viability decreased to 29.5%. LSOPC-nanoliposomes exhibited better protective effects against UVB irradiation than free LSOPC or vitamin C at concentrations of 12.5 μg/mL (Figure 4B) and 25 μg/mL (Figure 4C). Under 500 mJ/cm^2^ UVB irradiation, the cell viability with LSOPC-nanoliposomes increased significantly to 104.5% (12.5 μg/mL) and 108.5% (25 μg/mL), respectively (Figure 4C). Overall, the protective effect against exposure to UVB irradiation was in the following order: LSOPC-nanoliposomes > LSOPC > vitamin C. 

### 2.8. SOD and MDA Determination in UVB Injury Model

Superoxide dismutase (SOD) plays an important role in defending against photo-oxidative stress, which has been attributed to the strong free radical scavenging activity of this enzyme. Quantitative analysis of SOD levels is a good method to assess the oxidative damage status of cells [24].

**Figure 5:**
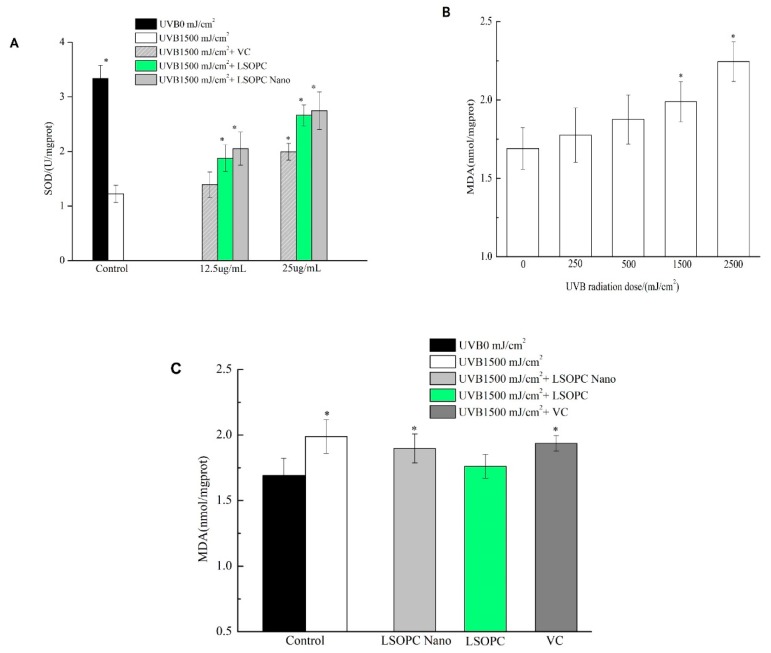
Superoxide dismutase (SOD) in HFF-1 cell before and after UVB irradiation with different concentrations of LSOPC, LSOPC Nano, and Vc, respectively (**A**); malonaldehyde (MDA) in HFF-1 cell under different doses of UVB radiation (**B**); MDA in HFF-1 cell before and after 1500 mJ/cm^2^ UVB irradiation with LSOPC, LSOPC Nano, and Vc (12.5 μg/mL), respectively (**C**). * *p* < 0.05.

As shown in Figure 5A, when compared with the control group (3.33 ± 0.24 U/mg protein), there was a significant decline of SOD levels in cells treated with UVB irradiation alone (1.22 ± 0.16 U/mg protein), which is indicative of severe cellular damage. At bioactive levels of both 12.5 and 25 μg/mL, the SOD levels increased in the following trend: vitamin C < free LSOPC <LSOPC-nanoliposomes. At a level of 12.5 μg/mL, the differences between the LSOPC samples and the controls were statistically significant, while that of the vitamin C group was not. For this reason, we chose this bioactive concentration to assess the different preventive effects of the three bioactive-treated groups on MDA formation. 

Lipid peroxide formation is associated with the oxidative damage of cells caused by UV irradiation, which changes membrane fluidity and influences membrane protein activity [25]. Malonaldehyde (MDA) is the major secondary metabolite of lipid peroxidation and is widely used as an indicator of cell membrane oxidative damage. As shown in Figure 5B, increasing the intensity of UVB irradiation significantly increased the MDA content in the cells. The MDA content became statistically different to the non-treated samples after exposure to 1500 and 2500 mJ/cm^2^ UVB irradiation. For this reason, a UVB irradiation of 1500 J/cm^2^ was chosen for the subsequent experiments.

According to Figure 5C, the levels of MDA in the cells treated with free LSOPC, LSOPC-nanoliposomes, or vitamin C were lower than that of the control group, suggesting that the degree of oxidative damage to the cells was decreased due to the antioxidant activity of the bioactive agents. At 12.5 μg/mL, free LSOPC showed some protection, with the levels of MDA formed (1.76 ± 0.09 nmol/mg protein) after UVB exposure being appreciably less than those in the control group (1.99 ± 0.13 nmol/mg protein). Conversely, there were no significant differences between the levels of MDA formed in the cells treated with LSOPC-nanoliposomes (1.89 ± 0.11 nmol/mg protein) or vitamin C (1.94 ± 0.06 nmol/mg protein) after UVB exposure compared to the control group. This result suggests that encapsulation of the LSOPC actually reduced its ability to reduce the MDA levels in the cells.

### 2.9. Protective Effect on Cell Viability under UVA Irradiation

**Figure 6:**
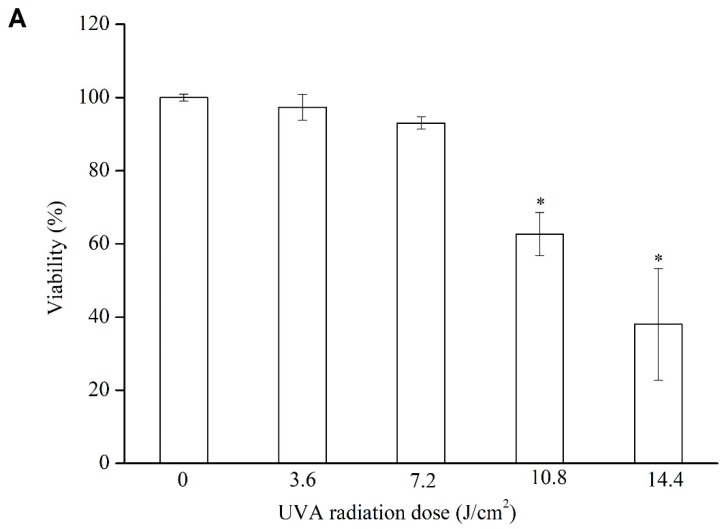

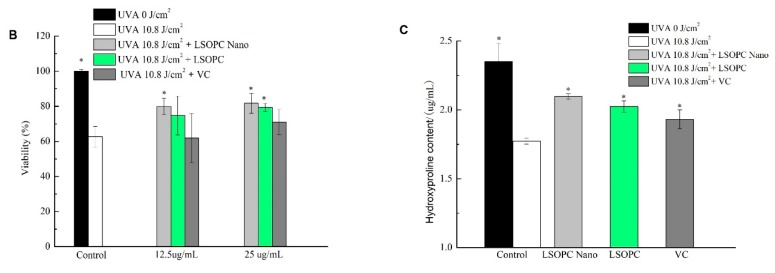
Cell viability of HFF-1 after different doses of UVA irradiation (**A**); protective effects of Vc, LSOPC, and LSOPC Nano on HFF-1 cell after 10.8 J/cm^2^ UVA irradiation (**B**); hydroxyproline formation before and after 10.8 J/cm^2^ UVA irradiation with LSOPC, LSOPC Nano, and Vc (12.5 ug/mL), respectively (**C**). * *p* < 0.05.

Even though the direct damage of UVA to skin is less than UVB, the penetrating power of UVA is stronger. Exposure to UVA irradiation affects the corium layer, particularly the internal formation of collagen. According to Figure 6A, cell viability progressively decreased as the intensity of UVA irradiation they were exposed to was increased. For instance, the cell viability decreased to 97.3% after exposure to 3.6 J/cm^2^ of UVA irradiation and to 38.0% after exposure to 14.4 J/cm^2^. For the subsequent experiments, a UVA irradiation of 10.8 J/cm^2^ (62.7% cell viability) was selected to treat the samples.

According to Figure 6B, the LSOPC-nanoliposome treatment provided the strongest protective effect against exposure to 10.8 J/cm^2^ UVA irradiation. For instance, at a bioactive level of 12.5 μg/mL, the cell viability was 80.0%, 74.7%, and 62.0%for LSOPC-nanoliposomes, free LSOPC, and vitamin C, respectively. Similarly, at a bioactive level of 25 μg/mL, the cell viability was 81.2%, 79.4%, and 70.7%, respectively. Overall, the protective effect under UVA irradiation was in the following order: LSOPC-nanoliposomes > free LSOPC > vitamin C, which was consistent with the UVB results.

### 2.10. Hydroxyproline Measurement in UVA Injury Model

Chronic UV irradiation increases epidermal thickness and promotes collagen loss, which leads to skin aging [26]. The degree of collagen loss can be assessed by measuring the hydroxyproline levels in the cells. According to Figure 6C, the hydroxyproline content in the cells exposed to 10.8 J/cm^2^ UVA irradiation (1.77 ± 0.02 μg/mL) was significantly lower than that of the untreated cells (2.35 ± 0.13 μg/mL). The hydroxyproline contents of the groups pretreated with12.5 μg/mL of LSOPC (2.02 ± 0.04 μg/mL), LSOPC-nanoliposomes (2.10 ± 0.02 μg/mL), and vitamin C (1.93 ± 0.07 μg/mL) were higher than that of the model group but still lower than that of the non-irradiated group. The content of the LSOPC-nanoliposomes treatment group was higher than that of LSOPC and vitamin C groups. This indicates that LSOPC-nanoliposomes had a better protective ability against collagen degradation and/or synthesis after UVA irradiation.

## 3. Materials and Methods 

### 3.1. Materials

Mature lotus seedpods of *Nelumbo nucifera* Gaertn. (cv.: Number 2 Wuhan plant) were harvested from Honghu District in Hubei province, China. Superoxide dismutase (SOD), malonaldehyde (MDA), and hydroxyproline assay kits were purchased from Nanjing Jiancheng Biology Engineering Institute (Nanjing, China). 3-(4,5-Dimethylthiazol-2-yl)-2,5-diphenyl tetrazolium bromide (MTT), monophenolase, l-DOPA, and l-tyrosine were purchased from Sigma Chemical Co. (St. Louis, MO, USA). Human foreskin fibroblast cell HFF-1 was purchased from the China Center for Type Culture Collection (CCTCC) (Wuhan, China).

### 3.2. Preparation of LSOPC Nanoliposomes

The extraction process of LSOPC was performed according to the published article of Wu et al. [8]. LSOPC nanoliposomes (LSOPC Nano) were produced using a reverse-phase evaporation method described in our previous studies [17]. Briefly, a mixture of cholesterol, soy lecithin (mass ratio 1:3, dissolved in chloroform), and LSOPC (0.33 mg/mL dissolved in 50 mmol/L PBS, pH = 7.4) was evaporated to remove the chloroform. Then, 30 mL of Tween 80 solution (16.7 mg/mL dissolved in PBS) was added to the mixture and evaporation was continued for another 20 min. The resulting mixture was then sonicated for 5 min (350 W, 5s/5s). According to our previous research [17], the mean particle size of the nanoliposomes prepared under these conditions was 35.57 ± 0.08 nm, with a polydispersity index value of 0.153 ± 0.01. The encapsulation efficiency was 71.97 ± 0.42%, achieved at the concentration of 1% of LSOPC. Additionally, transmission electron microscopy (TEM) confirmed the spherical vesicle structure of LSOPC nanoliposomes [17].

### 3.3. Residual Organic Solvent Measurement

Gas chromatography (6890N, Agilent) was performed to determine the residue of chloroform in the LSOPC Nano using an Agilent HP-5 column (30m × 0.32 mm, 0.5 μm) with a flame ionization detector (Palo Alto, CA, USA) [27]. The injection temperature was set at 170 °C and the detector temperature was set at 250 °C. The programmed temperature began at 35 °C for 2 min, then increased at a rate of 25 °C/min to 90 °C where it was held for 5 min, followed by an increase at a rate of 60 °C/min to 200 °C where it was held for 5 min.

### 3.4. Inhibition Effects of LSOPC Nano on the Tyrosinase Activity

#### 3.4.1. Effect of LSOPC on Monophenolase Activity

The measurement of monophenolase activity was conducted according to published articles with few modifications [4,28,29,30]. Briefly, 20 μL of LSOPC or LSOPC Nano at different concentrations were mixed with 100 μL of PBS. PBS (20 μL) was used as a blank control. Then, 10 μL of monophenolase (1 mg/mL) were added. Afterward, 40 μL of l-tyrosine were instantly added and vortexed for 5 min. The absorbance was measured at 475 nm every other minute. The experiments were performed in triplicate. The inhibition extent was expressed as the percentage necessary for 50% inhibition (IC50).

#### 3.4.2. Effect of LSOPC on Diphenolase Activity

The measurement of diphenolase activity was conducted according to published articles with few modifications [4,28,29,30]. Briefly, 150 μL of LSOPC or LSOPC Nano at different concentrations were mixed with 5 μL of monophenolase. The mixture was incubated at 25 °C for 10 min. Afterward, 30 μL of l-DOPA were instantly added. The absorbance value was measured at 475 nm. The experiments were performed in triplicate. The inhibition extent was expressed as the percentage necessary for 50% inhibition (IC50).

### 3.5. Fluorescence Quenching Analysis

#### 3.5.1. Fluorescence Assays of l-Tyrosine–LSOPC Interactions

These experiments were conducted using a fluorescence spectrophotometer (F-4600, Hitachi Limited, Tokyo, Japan) equipped with a 1.0 cm quartz cell according to a previously published article [31], with some slight modifications. The l-tyrosine concentration was fixed as 12 mmol/L, and LSOPC or LSOPC Nano systems were prepared with concentrations in the range from 25 to 500 μg/mL. After the l-tyrosine, PBS and LSOPC or LSOPC Nano were mixed evenly (1:3:1, *v*/*v*/*v*), and the mixture was incubated at 25 °C for 30 s. The excitation wavelength was set at 280 nm and the fluorescence quenching spectra were recorded between 300 and 450 nm. The excitation and emission slit widths were set at 5 nm.

#### 3.5.2. Fluorescence Assays of l-DOPA–LSOPC Interactions

The experiments were conducted by fluorescence spectrophotometer (F-4600, HIT) equipped with a 1.0 cm quartz cell according to a previously published article [31], with a few changes. The l-DOPA concentration was set as 0.5 mmol/L, and a series of LSOPC or LSOPC Nano dispersions were prepared with concentrations in the range from 25 to 500 μg/mL. After the l-tyrosine, PBS and LSOPC or LSOPC Nano were mixed evenly (1:3:1, *v*/*v*/*v*), and the mixture was incubated at 25 °C for 30 s. The excitation wavelength was set at 280 nm and the fluorescence quenching spectra were recorded between 300 and 450 nm. The excitation and emission slit widths were set at 5 nm.

### 3.6. Molecular Docking

The docking simulations were performed using Molecular Operating Environment (MOE), version 2018 (Chemical Computing Group, Montreal, QC, Canada). The 3D structure of tyrosinase protein was downloaded from the PDB database (PDB code: 6EI4) [32]. Before docking, both the receptor and the ligands were pretreated. The A chain of tyrosinase with active site (with two copper ions) was kept as receptor. The QuickPre operation was operated with the receptor. The ligands were built and energy-minimized using MOE. During the docking simulation, the following options were set: for the first scoring function, “London dG” was chosen for Rescoring 1 and its Retain option was dropped down to 30; second, “Forcefield” was chosen for refinement; third, the Rescoring 2 of the second scoring function was set to GBVI/WSA dG and Retain was set to 30. After docking, the conformation with the low S-score was chosen as the potential binding conformation [33].

### 3.7. Cell Culture

HFF-1 cell lines were maintained in DMEM growth medium supplemented with 15% fetal bovine serum (FBS), with added antibiotics penicillin (100 U/mL), streptomycin (100 μg/mL), and 1% sodium pyruvate. Cells were kept at 37 °C in a humidified atmosphere containing 5% CO_2_.

### 3.8. Cytotoxicity Assay

Cell viability was determined using an MTT assay [34]. First, 100 μL growth medium containing cells (2 × 10^5^/mL) were plated in 96-well plates. After 24 h incubation, cells were treated with various concentrations (0, 12.5, 25, 50, 100, and 200 μg/mL medium) of LSOPC or LSOPC Nano in FBS-free DMEM for 24 h. Each well was washed three times using 150 μL of PBS after removing the supernatant. Furthermore, 200 μL of serum-free MTT medium (0.5 mg/mL) were added to each well, followed by incubation for 4 h at 37 °C. Then, 150 μL of DMSO were added to each well after disposing the supernatant. Subsequently, the absorbance of each well was recorded at 490 nm using a Synergy HTX micro plate reader (BioTek, Burlington, VT, USA).

### 3.9. Protective Effects of LSOPC on HFF-1 Cell Exposed to Ultraviolet Radiation

#### 3.9.1. Cell Viability Assay

First, 100 μL growth medium containing cells (2 × 10^5^/mL) were plated in 96-well plates. After 24 h incubation, cells were treated with various concentrations (0, 12.5, and 25 μg/mL medium) of LSOPC, LSOPC Nano, or vitamin C in FBS-free DMEM for 12 h. Each well was washed three times using 150 μL of PBS after removing the supernatant. Furthermore, 100 μL of PBS were added to each well, followed by UVB or UVA irradiation. Irradiation was performed in PBS using a UVB lamp (850 μw/cm^2^) and a UVA lamp (1000 μw/cm^2^), respectively. After irradiation, PBS was removed and 100 μL of FBS-free DMEM were added, followed by 12 h incubation. The cell viability was measured using the MTT assay performed as described in the previous cytotoxicity assay.

#### 3.9.2. SOD and MDA Determination in UVB Injury Model

First, 2 mL growth medium containing cells (2 × 10^5^/mL) were plated in 6-well plates under the conditions mentioned above. Determination of MDA and SOD in cells was then performed using commercial assay kits (Nanjing Jiancheng Bioengineering Institute) [35].

#### 3.9.3. Hydroxyproline Measurement in UVA Injury Model

First, 2mL growth medium containing cells (2 × 10^5^/mL) were plated in 6-well plates under the conditions mentioned above. Determination of hydroxyproline in the cells was then performed using a commercial assay kit.

### 3.10. Statistical Analysis

Origin software 8.0 (Originlab Corporation, Northampton, MA, USA) was used to perform all statistical analyses. All data were calculated using one-way ANOVA of SPSS 17.0 (NICOM Intelligence, Mission Hills, CA, USA), followed by Tukey’s multiple-range test. Data were presented as means ± standard deviation (SD) and the statistical significance was defined as *p* < 0.05.

## 4. Conclusions

Overall, we used a variety of analytical tools to study the potential of an encapsulated plant-based bioactive agent (LSOPC) to protect against skin damage. Fluorescence spectroscopy showed that LSOPC-nanoliposomes possessed excellent inhibiting effects against both monophenolase and diphenolase activity. Additionally, encapsulated LSOPC exhibited a higher interaction affinity with the substrates of melanogenesis than free LSOPC. In the cell experiments, LSOPC-nanoliposomes showed better protective effects against UVB and UVA exposure than free LSOPC or vitamin C. SOD and MDA assays demonstrated that the protection mechanism may be related to the anti-photooxidation activity of the encapsulated bioactive agent. The hydroxyproline content measurements suggested that LSOPC-nanoliposomes have a better protective ability against collagen degradation and/or synthesis after exposure to UVA irradiation. Taken together, our results indicated that LSOPC-nanoliposomes can reduce the skin aging process and prevent UV damage, which suggests that they may be suitable for widespread application. Nevertheless, further studies are required using animals and humans to ensure their efficacy and safety. Moreover, studies are also required to ensure that they have the required optical, rheological, surface, and stability properties for utilization in commercial products.

## Supplementary Materials

The following are available online. Figure S1. GC chromatograms of chloroform and ethanol standard (**A**) chloroform:ethanol = 1:1; (**B**) chloroform:ethanol = 1:4; (**C**) LSOPC Nano.
