# Supplementary material for: Oligomeric Procyanidin Nanoliposomes Prevent Melanogenesis and UV Radiation-Induced Skin Epithelial Cell (HFF-1) Damage
Source: Molecules. 2020 Mar 24;25(6):1458. doi: 10.3390/molecules25061458 (PMC7145291; doi:10.3390/molecules25061458)
Supplement: Supplementary file 1 [file molecules-25-01458-s001.pdf]

Figure S1

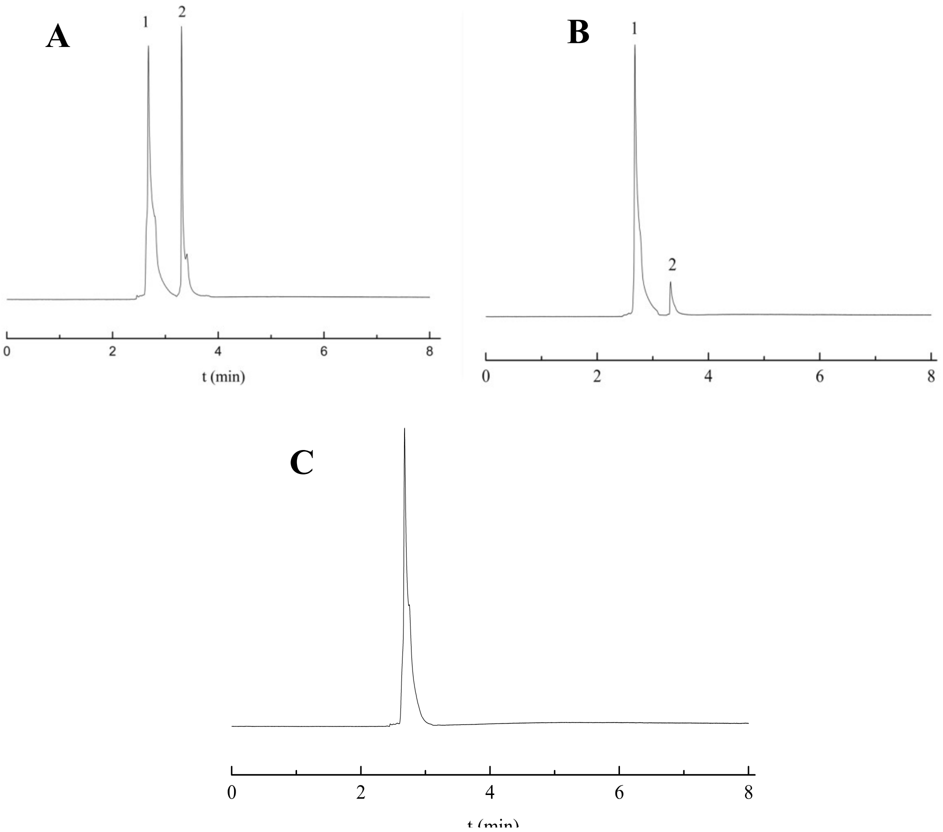

Figure S1. GC Chromatograms of chloroform and ethanol standard (A) chloroform: ethanol=1:1； (B). chloroform: ethanol =1:4; (C). LSOPC Nano.
